# Access to consciousness of briefly presented visual events is modulated by transcranial direct current stimulation of left dorsolateral prefrontal cortex

**DOI:** 10.1038/s41598-019-47527-4

**Published:** 2019-07-29

**Authors:** Stefano Sdoia, David Conversi, Anna Pecchinenda, Fabio Ferlazzo

**Affiliations:** Department of Psychology, Sapienza University, Rome, Italy

**Keywords:** Human behaviour, Consciousness

## Abstract

Adaptive behaviour requires the ability to process goal-relevant events at the expense of irrelevant ones. However, perception of a relevant visual event can transiently preclude access to consciousness of subsequent events — a phenomenon called attentional blink (AB). Here we investigated involvement of the left dorsolateral prefrontal cortex (DLPFC) in conscious access, by using transcranial direct current stimulation (tDCS) to potentiate or reduce neural excitability in the context of an AB task. In a sham-controlled experimental design, we applied between groups anodal or cathodal tDCS over the left DLPFC, and examined whether this stimulation modulated the proportion of stimuli that were consciously reported during the AB period. We found that tDCS over the left DLPFC affected the proportion of consciously perceived target stimuli. Moreover, anodal and cathodal tDCS had opposing effects, and exhibited different temporal patterns. Anodal stimulation attenuated the AB, enhancing conscious report earlier in the AB period. Cathodal stimulation accentuated the AB, reducing conscious report later in the AB period. These findings support the notion that the DLPFC plays a role in facilitating information transition from the unconscious to the conscious stage of processing.

## Introduction

The computational constraints of the human brain are experienced during everyday activities when multiple or fast-changing pieces of visual information must be managed concurrently or in rapid succession. Therefore, selection mechanisms are needed to ensure conscious access to task-relevant visual input at the expense of task-irrelevant input. Similarly, maintenance mechanisms are required to retain templates (e.g. task sets) that support the selection of task-relevant information for future mental operations. Most well-established cognitive models of consciousness functioning include mechanisms for gating (i.e. attention) and maintenance (i.e. working memory)^1–5^, but the processes and neural activity that facilitate the transition of information from unconscious to conscious stages remain unclear^6–8^.

One strategy for studying conscious access limitations involves overloading the visual attentional system by concurrently or rapidly presenting multiple task-relevant visual events, and assessing the probability of consciously reporting specific information according to the presence and/or temporal proximity of task-irrelevant information. In the Rapid Serial Visual Presentation (RSVP) procedure, visual events rapidly succeed one another in foveal vision at a rate of about ten items per second. The human ability to consciously recognize the second of two targets (T1 and T2) embedded among distractors is severely compromised if T2 is presented within 100–500 milliseconds after T1. Under these conditions, subjects perceive T2 at a rate of approximately 50%—a phenomenon referred to as the attentional blink (AB)^9–12^. On the other hand, T2 is generally reported when it immediately follows T1, which is termed the Lag-1 sparing effect^13,14^).

Notably, during the AB, the unreported T2 undergoes a high level of processing (e.g. semantic), indicating that AB is not due to resource limitations, but rather represents a failure of high-level representations to gain access to consciousness^15–23^. Both reported and missed targets produce similar semantic and repetition priming effects on consecutive successfully reported targets, and elicit event-related potentials (ERPs) associated with visual and semantic processing^18,19,22^. Neuroimaging studies demonstrate that missed visual stimuli have content-specific effects on the neural activity of functionally specialized visual areas^17,24^, indicating that even undetected events activate the associative cortex region involved in processing specific perceptual features. The AB reveals that perception of a relevant visual event is followed by a critical time window during which subsequent events cannot gain conscious access. Thus, the AB has attracted increasing interest in the fields of experimental psychology and cognitive neuroscience, as a suitable paradigm for studying gating mechanisms to consciousness and for identifying neural markers of conscious access^25^.

A broad network of frontal and parietal regions is implicated in the AB, with growing evidence suggesting that the dorsolateral prefrontal cortex (DLPFC) plays a more prominent role than parietal regions^17,24,26–31^. Notably, DLPFC neural activity discriminates between reported and unreported T2 during the AB window^17,27^, and greater DLPFC activity is associated with higher T2 detection rates^24^. London and Slagter (2015)^32^ recently reported that transcranial direct current stimulation (tDCS) over the DLPFC yielded a reduced AB in participants with a large initial AB size, and an increased AB in those with a small initial AB size. These findings suggest that prefrontal cortex activity may be a candidate neural marker for conscious access^33,34^. However, the causal role of the DLPFC in conscious access is far from being unambiguously established^35,36^, as other evidence suggests that conscious visual perception is primarily related to the activity of more posterior brain regions^37–43^. T2 detection during the AB also shows correlation with the activity of high-level occipito-temporal associative areas that are specialized in processing specific visual material^17,24,27^. Hence, further studies are needed to clarify the DLPFC’s contribution to conscious access during the AB.

Importantly, the predominant role of the DLPFC in AB has largely been inferred from brain imaging evidence, which enables recording of the metabolic indexes of neural activity, but has the limitation of being correlational. Greater insight into the DLPFC’s involvement in the AB can be obtained by actively manipulating the neural activity of this region and assessing the impact on conscious access of visual events. The non-invasive brain stimulation technique of tDCS allows transient modulation of spontaneous neuronal excitability through the delivery of a low constant electric current flow through two electrodes applied to the scalp. This electric current flow alters the polarization of the resting membrane potential, such that cortical excitability is increased by the anode electrode, and decreased by the cathode electrode^44–46^.

In the present study, we used tDCS to investigate the left DLPFC’s involvement in the conscious access of briefly presented visual events. In a sham-controlled experimental design, we applied anodal or cathodal tDCS to, respectively, potentiate or reduce the excitability of the left DLPFC during an attentional blink task. Our aim was to assess whether the proportion of target stimuli (T2) that could be consciously reported during the AB period was enhanced or impaired depending on the stimulation type.

## Methods

### Participants

The study participants included 34 right-handed healthy subjects, randomly assigned to one of two tDCS stimulation polarity conditions (cathodal and anodal, between-subject). The cathodal group included seventeen participants (10 women, mean age 24.2 years, and 7 men, mean age 21.9 years). The anodal group included seventeen participants (10 women, mean age 21.0 years, and 7 men, mean age 21.9 years). All participants reported normal or corrected-to-normal vision, no history of neurological or psychiatric disorders, and no ongoing medication use; they were naïve to the aims of the study. The sample size was defined through power analysis (using MorePower 6.0 software)^47^, using a medium-to-large partial eta^2^ of 0.2 for the highest order interaction, and a power of 0.9 to increase the chance of replicability. This partial eta^2^ was estimated from the Group × Lag interactions found in our previous behavioural studies (range: 0.18–0.23)^48,49^, as they used the same general methods used here, and the only prior study that investigated the effects of tDCS on the AB^32^ failed to report any group effects. All participants provided written informed consent. The present study was approved by the Ethics Committee of the Department of Psychology at Sapienza University, and was conducted in accordance with its policies.

### Attentional blink

Participants were individually tested in a dimly lit testing room. Stimuli were presented on a 17-inch Samsung computer monitor (refresh rate: 85 Hz) placed 60 cm from the participant’s head. The experiments were programmed in MATLAB 6.5, using Cogent 2000 Toolbox (developed at the FIL and the ICN) and Cogent Graphics (developed by John Romaya at the LON at the Wellcome Department of Imaging Neuroscience), on a computer running the Microsoft Windows XP operating system. Responses were recorded using a standard keyboard.

Participants performed a standard AB task. On each of 126 streams of stimuli, the two targets digits T1 and T2 (digits 1–9) were presented among 18 distractor letters (A, B, C, D, E, F, G, H, K, L, M, P, Q, R, S, T, U, X, Y, and Z), for 82 msec each. Each stimulus was immediately replaced by the next one, without masking. All stimuli were centrally presented in white on a grey background (approximately 115 cd/m^2^), subtending an approximate visual angle of one degree. On each stream, the target and distractor items were randomly selected with the constraints that T1 had to be different from T2, and the distractors had to be different from one another. T1 randomly appeared at serial position four or five, and T2 appeared at 82 (Lag 1), 247 (Lag 3), or 400 (Lag 5) msec after T1 (42 streams each). These three lags were applied with equal probability and in random order.

The total number of trials was chosen to be compatible with a 10-min tDCS stimulation period, and to reduce the chance of training effects^50^, or effects that reportedly modulate the basic AB, such as developing temporal expectancies and individual strategies to cope with the task^48–52^. Indeed, these factors are more likely with an increasing number of trials. Since we were interested in assessing the differential effect of anodal and cathodal tDCS on the AB, we used a number of trials that allowed detecting the typical AB while reducing the effect of other factors that modulate the basic AB effect.

Participants initiated each trial by pressing the space bar, and each stream started after 500 msec. At the end of each stream, participants reported the two targets, independently of the presentation order, without time pressure. Participants briefly practiced the task with 10 trials before starting each experimental session. No breaks were allowed once the experimental session was initiated.

### Transcranial direct current stimulation

A 1.5-mA direct current was induced by two saline-soaked circular sponge electrodes (diameter: 3 cm, density: 0.2 mA/cm^2^), and delivered by a battery-driven constant current stimulator (BrainStim E.M.S., srl Bologna, Italy) for 10 minutes during the AB task, with a 45-s fade in/fade out ramp. Current intensity and electrode size parameters were partly based on our previous work with tDCS^53^.

In two separate sessions one week apart, 17 participants underwent anodal and sham tDCS (anodal group), and 17 participants underwent cathodal and sham tDCS (cathodal group). The order of stimulation (active vs sham) was counterbalanced within the whole group. In the anodal group, nine participants received active stimulation during the first session and sham stimulation during the second session, and eight participants received sham stimulation during the first session and active stimulation during the second session. In the cathodal group, nine participants received sham stimulation during the first session and active stimulation during the second session, and eight participants received active stimulation during the first session and sham stimulation during the second session.

In the active stimulation condition, the anode or cathode was placed over the left dorsolateral prefrontal cortex (F3 according to 10–20 EEG International System), and the reference electrode was placed over the right orbitofrontal cortex (FP2). In the sham condition, electrode placement was the same but the stimulation included only the fade in/fade out phase and 2 seconds of stimulation. All participants were told they were receiving active stimulation. During the after-experiment debriefing, participants were informed on the theoretical and methodological aspects of the study, including that on one session they actually received sham stimulation. Participants were also explicitly asked whether they were able to say which session was which, and whether they noticed the difference between the two sessions. No participant reported to be able to recognize with certainty the active and the sham sessions. None of them reported different sensations between the two stimulation sessions. All participants completed the AB task before tDCS terminated.

### Statistical analyses

**Figure 1:**
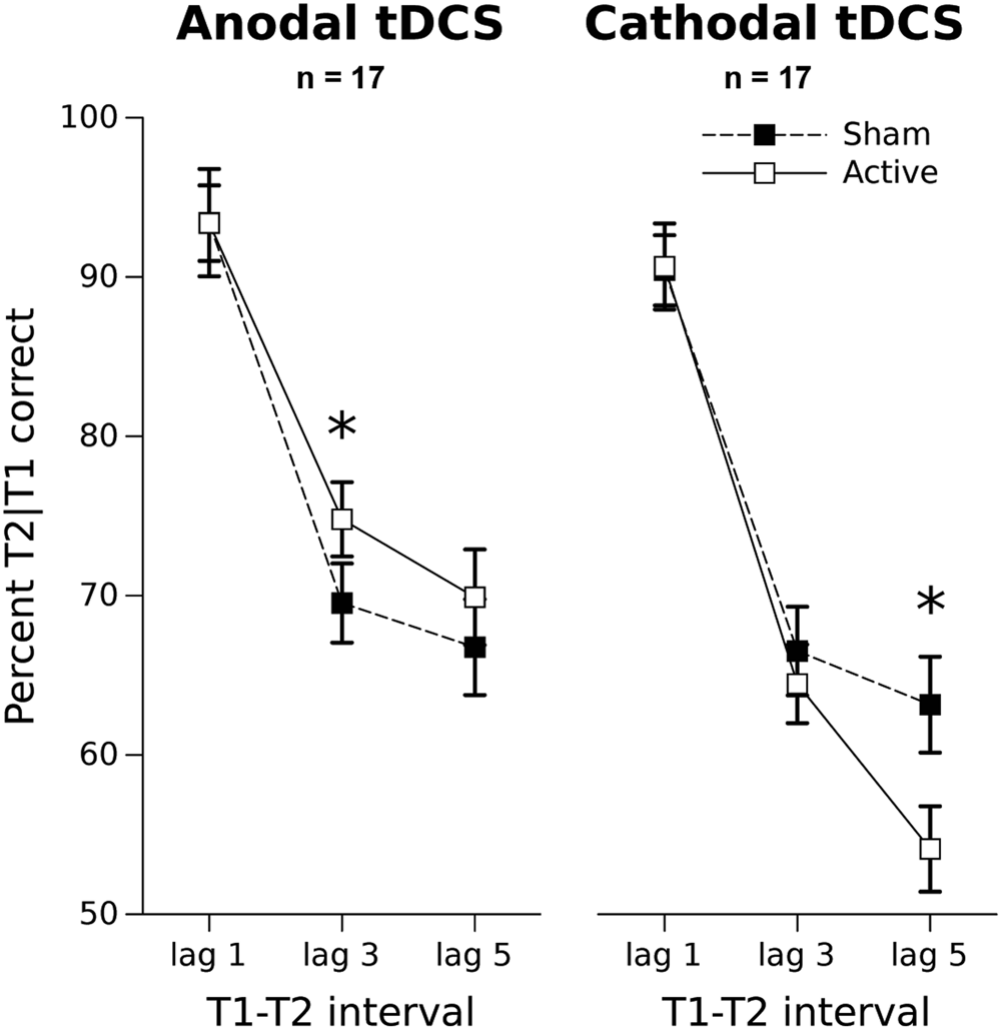
Mean percentage of T2 correct identifications, given T1 correctly recognized (T2|T1), as a function of the tDCS polarity (anodal and cathodal group), stimulation condition (Sham and Active) and lag (1, 3, 5). Bars denote standard errors according to Morey^84^ procedure for estimating the confidence intervals in within-subject designs.

For each experimental condition and lag, we computed the mean proportions of correctly reported T2 contingent upon correctly reported T1 (T2|T1) (Fig. 1). To test for between-group differences during sham stimulation, we used a 2 (Polarity: Anodal vs Cathodal, between factor) by 3 (Lag: 1, 3, 5, within factor) mixed ANOVA design on T2|T1. This enabled exclusion of any pre-existing between-group differences that might impact the effect of tDCS on the AB. We next tested the differential effects of the active stimulation condition using a 2 (Polarity: Anodal vs Cathodal, between factor) by 3 (Lag: 1, 3, 5, within factor) mixed factorial ANCOVA on T2|T1, including the accuracy averaged across the three lags during sham stimulation as a time-independent covariate. We chose a covariate approach because the AB is largely influenced by individual factors^54^. Using the sham condition as a covariate enabled comparison of the effects of anodal and cathodal tDCS on the AB, while controlling for individual differences in AB size during the sham session.

To assess the reliability of the above-described ANCOVA results, we ran two non-parametric bootstrap analyses^55,56^. In the first analysis, 400 bootstrap samples (N = 17) were randomly drawn with replacement separately from each group, and the same ANCOVA was run. We then computed the average F values (F_boot_) for each main effect and the interaction. A significant F_boot_ suggested that the original effect did not depend on the specific group composition in terms of participants. In the second analysis, all the data were pooled together, and 2000 bootstrap samples (N = 34) were randomly drawn with replacement from the data pool. Each bootstrap sample was then split into two samples, and the same ANCOVA was run. Due to the random drawing, all the null-hypotheses in the ANCOVA were true, and the empirical Fisher’s F distribution was used to estimate the probability (p_boot_) of an F value equal to or greater than the observed F value under the null-hypothesis. A p_boot_ of <0.05 suggested that the original effect did not result from small deviations from the assumptions underlying the ANCOVA, undetected at the specific tests.

To further assess the effect of tDCS upon the AB, we directly compared T2|T1 accuracy under active (anodal or cathodal) and sham stimulation, as a function of Lag, in a Polarity (anodal vs cathodal, between-subject) by Stimulation (Active vs Sham, within-subject) by Lag (1, 3, 5) ANOVA design. A standard alpha level of 0.05 was set for all the statistical analyses.

## Results

The Levene’s test for homogeneity of variance, and the Box M test for equality of covariance matrices, yielded no significant departures from the assumptions underlying the ANOVA tests (p > 0.19 in all cases). An ANOVA on the participants’ age as a function of Gender and Stimulation polarity failed to show any significant effect (Gender: F(1,30) = 0.16, p = 0.7; Polarity: F(1,30) = 0.72, p = 0.40; Gender by Polarity: F(1,30) = 0.72, p = 0.40). The ANOVA on T2|T1 accuracy during sham stimulation demonstrated a significant effect of Lag (F(2,64) = 51.48, p = 0.0001, η_p_^2^ = 0.62), revealing the typical AB, but no significant Polarity or Polarity × Lag interaction (F < 1 in both cases), indicating no between-group differences in the AB effect under sham condition. However, individual variations in AB magnitude could still influence the tDCS effects^32^. Therefore, in the critical analysis of T2|T1 accuracy, we included the accuracy at the three lags during sham stimulation as a time-independent covariate. We found that this covariate affected the dependent variable (T2|T1 accuracy during anodal and cathodal stimulation) at both Lag 1 (F(2,58) = 9.24, p = 0.00032, η_p_^2^ = 0.24) and Lag 5 (F(2,58) = 11.43, p = 0.00006, η_p_^2^ = 0.28), suggesting that the effects of tDCS on AB partly depended on the participant’s AB magnitude in the sham condition. With the accuracy during sham stimulation included as a covariate, the ANCOVA removes the effects that are dependent on the participant’s performance during the sham stimulation. The ANCOVA showed a significant main effect of Polarity (F(1, 29) = 12.46, p = 0.00141, p_boot_ = 0.003, η_p_^2^ = 0.30, F_boot_ (1, 29) = 16.26, p = 0.008), revealing tDCS polarity-specific effects on overall T2|T1 accuracy, with a larger proportion of T2|T1 identifications during anodal tDCS compared to cathodal tDCS. Importantly, this main effect was further qualified by a significant Polarity × Lag interaction (F(2, 58) = 4.99, p = 0.01526, p_boot_ = 0.008, η_p_^2^ = 0.13, F_boot_ (2, 58) = 5.97, p = 0.035), indicating that anodal and cathodal tDCS had different effects on performance over the three lags. Specifically, anodal and cathodal stimulation did not affect T2|T1 accuracy at Lag 1 (p = 0.20014 Fisher LSD; Table 1). Conversely, the T2|T1 proportion was significantly larger during anodal compared to cathodal stimulation at both Lag 3 (p = 0.00246 Fisher LSD) and Lag 5 (p = 0.000008 Fisher LSD), indicating that anodal stimulation over the left DLPFC yielded a smaller AB effect. These findings indicated that anodal and cathodal tDCS differentially affect T2|T1 accuracy, even when individual differences in AB size during sham stimulation were accounted for by the ANCOVA.

**Table 1:** Percent T2|T1 correct and standard errors as a function of stimulation polarity (anodal/cathodal), stimulation condition (sham/active) and T1-T2 interval (lag 1, lag 3, lag 5).

Polarity	Stimulation condition	T1-T2 interval	Percent T2|T1 correct	SE
Anodal tDCS	Sham	Lag 1	93.41	3.37
		Lag 3	69.54	2.48
		Lag 5	66.77	3.01
	Active	Lag 1	93.38	2.37
		Lag 3	74.79	2.33
		Lag 5	69.90	3.00
Cathodal tDCS	Sham	Lag 1	90.42	2.20
		Lag 3	66.52	2.78
		Lag 5	63.16	3.01
	Active	Lag 1	90.66^*^	2.70^*^
		Lag 3	64.47	2.47
		Lag 5	54.10	2.68

^*^After having removed the outlier value at Lag 1 of the active condition from one participant in the cathodal group (more than 3 SD below the group mean) and replaced it by the group mean, the percent of T2|T1 correct at lag 1 on the Cathodal tDCS condition is 89.12 with a SE of 2.31.

Standard errors were computed according to Morey (2008)^84^ procedure for estimating the confidence intervals in within-subject designs.

In the Polarity (anodal vs cathodal, between-subject) by Stimulation (Active vs Sham, within-subject) by Lag (1, 3, 5, within-subject) ANOVA, T2|T1 proportion from the active condition at Lag 1 from one participant in the cathodal group was found to be more than 3 standard deviations below the group mean, and thus was replaced by the group mean^57^. Results of the analysis showed a significant main effect of Lag (F(2,64) = 75.536, p < 0.0001, η_p_^2^ = 0.7), a significant Stimulation by Polarity interaction (F(1,32) = 4.45, p = 0.04282, η_p_^2^ = 0.12) and, most importantly, a significant second order Stimulation by Polarity by Lag interaction (F(2,64) = 3.314, p < 0.04269, η_p_^2^ = 0.09). This last interaction has been decomposed by post-hoc testing separately for the anodal and cathodal groups. In the anodal group, the T2|T1 accuracy at Lag 1 during the active stimulation did not differ from the accuracy during the sham stimulation (p = 0.98976 Fisher LSD). At Lag 3, anodal tDCS significantly improved the T2|T1 proportion compared to the sham condition, yielding a reduced AB (p = 0.03497 Fisher LSD). Instead, no difference emerged between anodal and sham tDCS conditions at Lag 5 (p = 0.20374 Fisher LSD).

In the cathodal group, the T2|T1 accuracy at Lag 1 during the active stimulation did not differ from the accuracy during the sham stimulation (p = 0.92170 Fisher LSD). We observed no differential effects on T2|T1 identification between cathodal and sham tDCS conditions at Lag 3 (p = 0.40215 Fisher LSD). Conversely, cathodal tDCS significantly reduced the T2|T1 proportion at Lag 5, exacerbating the AB compared to sham stimulation (p = 0.0004 Fisher LSD). Hence, within the AB time window we explored, anodal and cathodal tDCS had opposite effects on T2|T1 accuracy, with anodal tDCS attenuating the AB at Lag 3, while cathodal tDCS exacerbated the AB at Lag 5 but not at Lag 3.

As previously mentioned, London and Slagter (2015)^32^ recently reported that individual variability in AB size interacts with the effects of anodal tDCS over the DLPFC, reducing the AB in participants with a large AB, and increasing the AB in participants with a small AB. Notably, in their study, the effects of anodal tDCS on AB did not appear at the group level, and cathodal tDCS did not affect AB at the group or individual level. Thus, the authors suggested that the effects of anodal tDCS exclusively depended on the individual’s AB magnitude^32^. In contrast, in our present study, the effect of anodal tDCS on AB at Lag 3 was not dependent on individual differences in AB magnitude measured during sham session. Rather, we found that anodal tDCS reduced the AB even when accounting for individual differences in AB size during the sham session. Nonetheless, it remained possible that the tDCS-induced AB reduction at Lag 3 could be of a greater magnitude in individuals with a large AB size. To further assess whether individual AB level during sham stimulation influenced the effects of tDCS, and to facilitate a clear comparison with findings of London and Slagter (2015)^32^, we examined correlations between individual AB magnitude during sham stimulation and the changes induced by anodal and cathodal tDCS, using the same procedure as London and Slagter (2015)^32^. AB magnitude was calculated as the largest difference in T2|T1 identifications between Lag 1 and the following lags for each stimulation condition. Results revealed that AB magnitude during sham stimulation had a significant inverse relationship with the change in AB magnitude during anodal tDCS (r = −0.53; p = 0.02928), but not with the change in AB magnitude during cathodal tDCS (r = −0.34, p = 0.179754), as also London and Slagter (2015)^32^ reported. However, the correlation between the AB magnitude during the sham stimulation (X) and the change induced by the active stimulation (Y-X) suffers of two methodological concerns known as mathematical coupling and regression to the mean effects, that may yield a 0.71 correlation even when no actual relationship exists^58^. To solve the problem of spurious correlation between an initial value and its change after treatment, Oldham (1962)^59^ suggested a different correlation equation that is independent of mathematical coupling and regression to the mean effects. We used the Oldham’s strategy to further assess the reliability of the correlation between AB size measured during sham stimulation and its change after anodal and cathodal tDCS. Our results showed that the correlations between the AB magnitudes measured during sham and anodal tDCS and between AB magnitude measured during sham and cathodal tDCS, according to the Oldham (1962)^59^ equation, were not significant (−0.33, p = 0.191 for the anodal tDCS and 0.08, p = 0.750 for the cathodal tDCS), indicating that the changes induced by anodal and cathodal tDCS did not depend on the magnitude of the AB measured during sham stimulation.

## Discussion

Here we investigated the DLPFC’s involvement in the conscious access of brief visual events by applying tDCS during an AB task, and examining whether the proportion of consciously reported target stimuli during the blink period changed depending on tDCS polarity over the left DLPFC. Our main finding was that tDCS over the left DLPFC modulated the targets’ access to consciousness during the AB period. Anodal tDCS improved T2 perception, reducing the AB when T2 occurred 247 msec after T1 (Lag 3). In contrast, cathodal tDCS hindered T2 perception, increasing the AB when T2 occurred 400 msec after T1 (Lag 5).

The present findings correspond to previous neuroimaging evidence indicating that the prefrontal cortex plays a role in conscious access during the AB^17,24,26–28^, and extend previous knowledge by providing evidence of the DLPFC’s involvement in gating stimulus access to consciousness during the blink period. Moreover, our results show that the effects of anodal and cathodal tDCS over the left DLPFC follow different temporal patterns. Anodal stimulation improved stimulus access to consciousness earlier in the AB period (Lag 3), while cathodal stimulation impaired stimulus access to consciousness later in the AB period (Lag 5). A possible, albeit speculative, explanation for the differential effects of anodal and cathodal tDCS is that cathodal and anodal tDCS may have affected the excitability of different neuronal populations within the DLPFC. For example, in the primary motor cortex, anodal tDCS decreases GABA levels, while cathodal tDCS affects glutamate levels^60^. Accordingly, administration of the GABA agonist lorazepam modulates the effects of anodal but not cathodal stimulation^61^. Moreover, fMRI studies suggest that anodal and cathodal stimulation modulate distinct systems-level networks within the active motor system^62^.

Our results add to the findings of London and Slagter (2015)^32^, who did not describe group effects depending on stimulation type, and who reported that anodal tDCS induced a reduced AB in participants with a large initial AB size, and an increased AB in participants with a small initial AB size. In our present study, we found that anodal tDCS attenuated the AB, whereas cathodal tDCS accentuated the AB. Compared to the study of London and Slagter (2015)^32^, our present study used shorter tDCS sessions, smaller electrodes, and stronger stimulation. Another important difference is that in London and Slagter’s study, the target were defined by the colour, which was the only feature differentiating targets (coloured letters) from distractors (white letters). Colour identification calls upon perceptual-driven attentional selection processes (i.e. bottom-up attentional capture) for target selection during the RSVP. In contrast, in our present study, targets and distractors shared the same colour and the target items were instead defined by stimulus identity (target numbers vs distractor letters). Thus, in our study, target selection largely relied on top-down attentional processes, and did not require perceptual-driven attentional selection. Top-down control of attention is mostly related to PFC activity, while the exogenous component is more related to the activity of posterior regions^63^. Accordingly, tDCS over the PFC would have a greater effect on the top-down component of attentional selection, and a lesser effect on the bottom-up component, contributing to the differences between the two studies. Another relevant difference relates to the experimental design. London and Slagter (2015)^32^ employed a pre/during/post-stimulation design (repeated for two sessions), which required participants to be involved in the task during a large number of trials. Training for up to one hour reportedly reduces the AB, and this effect can persist over time^50^. The large number of trials may have attenuated the effect of tDCS on the AB in London and Slagter’s study. Therefore, in the current study, the relatively short duration of the task, achieved by using a sham-based within-subject control condition, together with target selection dependent on top-down factors may have facilitated the detection of differences in the AB engendered by anodal and cathodal tDCS over the left DLPFC.

Since in the anodal group there were fewer participants who completed the anodal session second, as opposed to the sham condition second, one may wonder whether the order of stimulation affected the results. However, if practice reduced the attentional blink this imbalance is conservative as it would have worked against the reduction in the attentional blink by anodal stimulation in the participants who completed sham second. Similarly, in the cathodal group there were more participants who completed cathodal second, so again conservative given that the attenuating effects of practice on the attentional blink would have worked against the accentuating effects of cathodal stimulation. Also, a differential practice effect should have emerged in the analysis on the sham condition data, but this analysis failed to show any group differences or interaction between group and lag.

One limitation of our present study was that we did not assess performance with lags of longer than 400 msec. Thus, we could not evaluate any potential effects of tDCS on AB duration, or on recovery from the AB. Indeed, it appeared that cathodal tDCS impacted the AB at 400 msec after T1 presentation (i.e. Lag 5). Moreover, in the sham condition, the performance at Lag 5 suggested that the AB could have not completely reached its peak at Lag 3, since T2 accuracy did not seem to be recovering yet at Lag 5. While unusual, this is not entirely surprising, considering that Lag 5 occurred at 400 msec after T1, which was well within the AB window of 500 msec. Another limiting factor of the current study was that we did not directly assess on-line changes in DLPFC neural activity during tDCS. Since both anodal and cathodal electrodes have current flowing through them it is possible that the effects we found were also related to changes in the neural activity of the region below the reference electrode or other cortical regions^64^.

The mechanism by which the DLPFC modifies gating stimulus access to consciousness during the AB remains to be determined. The DLPFC has been implicated in target stimuli maintenance and consolidation in working memory against distracting stimuli^65,66^. The DLPFC may increase the duration of fleeting sensory information by sustaining activity in feature-specific posterior regions representing the current memory targets^66^ and/or by suppressing activity in distractor-related regions^67^. For example, cathodal tDCS over the DLPFC reportedly reduces distractor interference in a Face-Word Interference task via bottom-up degradation of neural signals^53^. When T2 stimuli consist of scene targets, the DLPFC’s activity for conscious report of T2 during the AB is accompanied by greater activity in the medial associative temporal cortex involved in high-level scene representations (the parahippocampal place area)^17,24^. However, other studies have reported a negative correlation between T2 detection during the AB and activity in occipitotemporal areas specialized in processing specific visual material^27,29^. Indeed, recent findings challenge the notion that the AB is due to a failure of target consolidation in working memory^68–71^. Studies have demonstrated that multiple consecutive targets can be successfully reported as long as there are no intervening distractors^72,73^. Moreover, the AB can be attenuated by a concurrent secondary task^74^, by reducing the subject’s focus on target identification^75^, by a change in task instruction^48,49^, by task-irrelevant visual motion or flicker^76^, and by probabilistic properties and temporal expectancy during the RSVP^52^. Importantly, electrophysiological evidence indicates that working memory consolidation occurs even for missed T2, but that this consolidation concerns the items following T2 (e.g. distractors) during the RSVP^77^, suggesting that the AB may be due to target mis-selection rather than the decay of target representation in working memory. Based on these findings, theories of AB have been developed that emphasize the contribution of gating mechanisms (e.g. attentional selection) rather than consolidation in working memory^78,79^ but see also^80,81^. It has been suggested that the DLPFC hosts neuronal populations that source attentional gate signals^82^ and, together with the basal ganglia, may represent a neural system for enhancement of task-relevant and suppression of distracting information^83^. tDCS over the left DLPFC may influence the activity of the prefrontal/basal ganglia network, facilitating or hindering the selection of correct items.

In conclusion, our present results provide evidence that tDCS over the left DLPFC induced changes in the efficiency of conscious report of rapidly presented visual events during the attentional blink period. These findings may suggest that the DLPFC plays a role in allowing the transition of information from unconscious to conscious stages of processing. It remains unclear whether the DLPFC supports this transition through gating, working memory maintenance, or consolidation processes. Future research should investigate whether DLPFC activity is sufficient for conscious perception, or whether conscious access during the AB requires widespread recurrent processing from the DLPFC to posterior high-level visual areas and/or interactions with subcortical structures.

## Supplementary information

T1 accuracy
