# Supplementary material for: Access to consciousness of briefly presented visual events is modulated by transcranial direct current stimulation of left dorsolateral prefrontal cortex
Source: Sci Rep. 2019 Jul 29;9:10950. doi: 10.1038/s41598-019-47527-4 (PMC6662720; doi:10.1038/s41598-019-47527-4)
Supplement: Supplementary file 1 — T1 accuracy [file 41598_2019_47527_MOESM1_ESM.pdf]

Supplementary Information

Title:

Access to consciousness of briefly presented visual events is modulated by transcranial direct current stimulation of left dorsolateral prefrontal cortex

Authors:

Stefano Sdoia*, David Conversi, Anna Pecchinenda and Fabio Ferlazzo

Department of Psychology, Sapienza University, Rome, Italy

In order to rule out the hypothesis that the tDCS effects did not target selectively the T2|T1 accuracy but influenced also the T1 accuracy itself, we investigated the effects of tDCS on T1 identification (Table S1). We ran the same analyses used for T2 performance: a mixed factorial ANCOVA design [Stimulation: Anodal vs Cathodal (between factor) × Lags: 1, 3, 5 (within factor)] on T1, including T1 accuracy at the three lags during sham stimulation as a time-independent covariate. The results did not indicate significant main effects of Lag ($F(2,58) = 0.11$, $p = 0.89739$) or Stimulation ($F(1,29) = 3.29$, $p = 0.08012$), or a significant Lag × Stimulation interaction ($F(2,58) = 0.37$, $p = 0.69311$). We also did not find that the covariate interacted with the other factors at Lag 1 ($F = 1.10$, $p = 0.33728$), Lag 3 ($F = 0.22$, $p = 0.80329$), or Lag 5 ($F = 0.53$, $p = 0.59189$).

TABLE S1. Percent T1 correct and standard errors as a function of stimulation polarity (anodal/cathodal), stimulation condition (sham/active) and T1-T2 interval (lag 1, lag 3, lag 5). Standard errors were computed according to Morey (2008)[60] procedure for estimating the confidence intervals in within-subject designs.

Polarity	Stimulation condition	T1-T2 interval	Percent T1 correct	SE
Anodal tDCS	Sham	Lag 1	79.13	1.62
		Lag 3	88.52	1.35
		Lag 5	89.36	1.53
	Active	Lag 1	82.77	1.88
		Lag 3	90.90	1.31
		Lag 5	90.48	1.16
Cathodal tDCS	Sham	Lag 1	74.05	2.24
		Lag 3	80.88	2.07
		Lag 5	79.00	2.20
	Active	Lag 1	76.89	2.04
		Lag 3	83.19	1.90
		Lag 5	84.45	1.66
